# RETRACTION: Graphene‐Based MicroRNA Transfection Blocks Preosteoclast Fusion to Increase Bone Formation and Vascularization

**DOI:** 10.1002/advs.75901

**Published:** 2026-05-27

**RETRACTION**: C. Dou, N. Ding, F. Luo, T. Hou, Z. Cao, Y. Bai, C. Liu, J. Xu, and S. Dong, “Graphene‐Based MicroRNA Transfection Blocks Preosteoclast Fusion to Increase Bone Formation and Vascularization,” *Advanced Science* 5, no. 2 (2018): 1700578, https://doi.org/10.1002/advs.201700578.

The above article, published online on 04 December 2017 in Wiley Online Library (wileyonlinelibrary.com), and has been retracted by agreement between the journal Editor‐in‐Chief, Kirsten Severing; and Wiley‐VCH GmbH. Concerns were raised by a third party on PubPeer [1] that images in Figure 3c contained overlapping sections. A correction [2] was published on 04 August 2021 to address these concerns. Subsequently, additional concerns were raised by another party on PubPeer [3] that the “0” image in Figure 3b overlapped with an image published in another article by some of the same authors [4]. Both articles underwent peer review at the same time, and each article reports different experimental conditions.

Further investigation by the publisher found that the originally published “RANKL(‐)” and “50” images in Figure 3c (which were replaced by the correction published in 2021) had also been previously published in another article by some of the same authors [5]. Additionally, the investigation found evidence that a portion of the Actin band in Figure 4d had also been reported as a GAPDH band in Figure 4D in Dou et al. 2024 [4].

The authors responded to an inquiry by the publisher. While the authors shared data and images corresponding to the experiments reported in this article, the publisher found that there were further discrepancies in the original data and that the authors did not provide a satisfactory explanation for data re‐use across multiple publications.

Following a review of this data, all parties have concluded that the evidence of image overlap with other articles casts doubt on the experimental results reported in this article. Furthermore, while the correction published in 2021 was intended to resolve concerns of image overlap within this article, the further evidence that the originally published images in Figure 3c had been reported as different samples in an earlier article adds further concerns about the authenticity of the experimental results.

Because the publisher and the editors have lost confidence in the data and results, the article must be retracted. The authors were informed of the retraction.
